# Mouse T-cells restrict replication of human immunodeficiency virus at the level of integration

**DOI:** 10.1186/1742-4690-5-58

**Published:** 2008-07-08

**Authors:** Hanna-Mari Tervo, Christine Goffinet, Oliver T Keppler

**Affiliations:** 1 Department of Virology, University of Heidelberg, Heidelberg, Germany

## Abstract

**Background:**

The development of an immunocompetent, genetically modified mouse model to study HIV-1 pathogenesis and to test antiviral strategies has been hampered by the fact that cells from native mice do not or only inefficiently support several steps of the HIV-1 replication cycle. Upon HIV-1 infection, mouse T-cell lines fail to express viral proteins, but the underlying replication barrier has thus far not been unambiguously identified. Here, we performed a kinetic and quantitative assessment of consecutive steps in the early phase of the HIV-1 replication cycle in T-cells from mice and humans.

**Results:**

Both T-cell lines and primary T-cells from mice harbor a severe post-entry defect that is independent of potential species-specTR transactivation. Reverse transcription occurred efficiently following VSV-G-mediated entry of virions into mouse T-cells, and abundant levels of 2-LTR circles indicated successful nuclear import of the pre-integration complex. To probe the next step in the retroviral replication cycle, i.e. the integration of HIV-1 into the host cell genome, we established and validated a nested real-time PCR to specifically quantify HIV-1 integrants exploiting highly repetitive mouse *B1 *elements. Importantly, we demonstrate that the frequency of integrant formation is diminished 18- to > 305-fold in mouse T-cell lines compared to a human counterpart, resulting in a largely abortive infection. Moreover, differences in transgene expression from residual vector integrants, the transcription off which is cyclin T1-independent, provided evidence for an additional, peri-integrational deficit in certain mouse T-cell lines.

**Conclusion:**

In contrast to earlier reports, we find that mouse T-cells efficiently support early replication steps up to and including nuclear import, but restrict HIV-1 at the level of chromosomal integration.

## Background

Human immunodeficiency virus type 1 (HIV-1) displays a highly restricted host and cell tropism and is only capable of efficient replication in primary and immortalized T-cells and macrophages of human origin. Cells from native mice do not or only inefficiently support various steps of the HIV-1 replication cycle [1-7]. The precise mapping of some of these species-specific barriers has, on one hand, facilitated the identification and molecular characterization of critical host factors, and, on the other hand, highlighted the complexity of the task to develop genetically altered mice that are fully permissive for HIV-1 infection.

The by far most prominent category of barriers thus far identified in mouse cell lines appears to be recessive in nature. Blocks in this category are characterized by an inability of mouse orthologues of cellular proteins that are essential cofactors for HIV-1 replication in human cells to support distinct replication steps of the virus. HIV-1 entry provides a compelling example since CD4 and the chemokine co-receptor CCR5 from mice bind the HIV-1 envelope glycoprotein with presumably only low affinity and this interaction is insufficient to support virion fusion [4,5,8]. Moreover, the discovery that expression of the human HIV-1 receptor complex largely overcomes the entry restriction has provided the rationale for the development of permissive multi-transgenic mouse and rat models through a block-by-block humanization [9]. Along these lines, expression of the human version of the Tat-interacting protein cyclin T1 was shown to boost HIV-1 transcription in mouse cells *in vitro *and *in vivo *[3,7,10-14]. Additional, less-defined blocks in the late phase of the HIV-1 replication cycle in NIH3T3 cells add up to a profound drop in the yield of viral progeny (up to 10^4^-fold) from a single round of replication [4,5,15]. Also these late-stage barriers in mouse fibroblasts display a recessive phenotype and likely result from non-functional mouse cofactors since they can be surmounted in mouse-human heterokaryons [4,5,15-17].

Cellular restriction factors, defining a different class of barrier characterized by dominant inhibitory activities, can interfere with lentiviral replication in a species-specific manner. Of potential relevance in the rodent context, the incorporation of the cytidine deaminase APOBEC3G of mouse origin into particles cannot, in contrast to its human orthologue, be counteracted by the HIV-1 Vif protein, resulting in a pronounced reduction in particle infectivity [18]. Providing another example, an early post-entry barrier has been reported for a SIVmac reporter virus in NIH3T3 cells, which displayed typical characteristics of a restriction factor [19].

However, most of these replication barriers in mice have been described in fibroblast cell lines and the efficiency of different steps of the HIV-1 replication cycle in more relevant target cells has remained elusive. More recently, a severe post-entry defect has been reported in infected mouse T-cells [19-21]. One study mapped this defect to a reduced efficiency of reverse transcription and nuclear import of the HIV-1 pre-integration complex [20]. A second study, in contrast, suggested nuclear import to be the sole cause of the early-phase restriction [21].

Here, we performed a kinetic and quantitative assessment of consecutive steps in the early phase of the HIV-1 replication cycle in T-cells from mice and humans. Starting from a single viral challenge, the efficiency of virus entry, reverse transcription, nuclear import, the frequency of integration, as well as transgene expression off a cytomegalovirus (CMV) immediate early promoter or off the HIV-1_NL4-3 _LTR were carefully monitored to pinpoint the restriction.

## Results

### HIV-1-infected mouse T-cell lines do not express a CMV-driven GFP reporter despite efficient virion entry

We first sought to establish a quantitative relationship between the ability of HIV-1 virions to enter T-cells of mouse and human origin and, subsequently, to express a reporter gene in these target cells. To ensure comparable conditions in the cross-species comparisons, we employed an HIV-1 based lentiviral vector encoding for GFP driven by a cytomegalovirus immediate early promoter (HIV-CMV-GFP), which was pseudotyped with the vesicular stomatitis virus glyco-protein (VSV-G). Notably, the expression of GFP from this vector is not influenced by HIV-1 Tat/cyclin T1-dependent, potentially species-specific differences in LTR transactivation [3]. Through incorporation of enzymatically active β-lactamase-Vpr fusion proteins (BlaM-Vpr) during virus production the efficiency of HIV-1 entry into target cells was specifically measured by CCF2 substrate cleavage in a flow cytometry-based virion-fusion assay [22,23].

**Figure 1:**
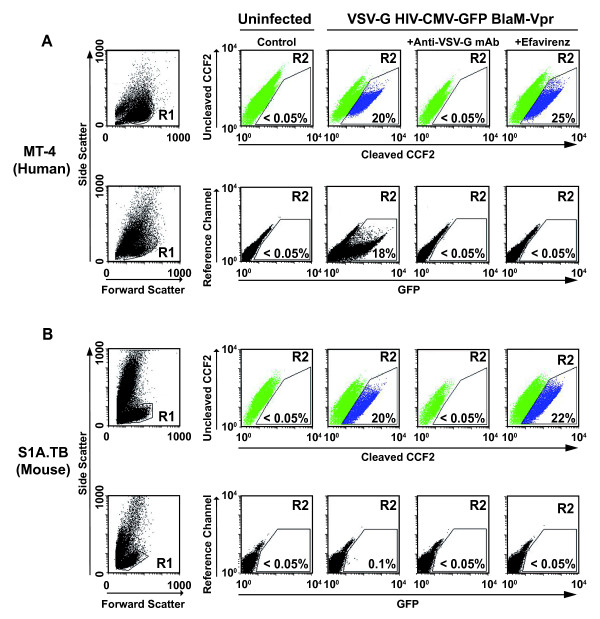
**Mouse T-cells do not support CMV-driven reporter gene expression following VSV-G-mediated virion entry**. Fusion of the VSV-G pseudotyped lentiviral vector carrying BlaM-Vpr (VSV-G HIV-CMV-GFP BlaM-Vpr) and subsequent GFP reporter gene expression was analyzed in (A) human MT-4 and (B) mouse S1A.TB T-cell lines by flow cytometry 6 h and 3 d p.i., respectively. Cells were challenged with the VSV-G pseudotyped vector either in the presence of the neutralizing anti-VSV-G monoclonal antibody I1, the NNRTI efavirenz, or left untreated. Shown are representative FACS dot plots of viable T-cells (gate R1; left panels) for the detection of the cleaved CCF2 substrate (gate R2; blue color; upper panels in A and B), reflecting HIV-1 entry, or early CMV-driven GFP expression (gate R2, lower panels in A and B). The relative percentage of cells in R2 is given.

Following a single challenge with this dual HIV-1 reporter virus, T-cell lines of human and mouse origin were analyzed for virion fusion and early gene expression, 6 h p.i. and on day 3 p.i., respectively. Fig. 1 depicts representative flow cytometric data of both of these analyses for MT-4 (human) and S1A.TB (mouse) T-cells, in which gate R2 defines the cleaved CCF2 (blue fluorescence emission)-positive subpopulation (Fig. 1A, B; upper panels) or the GFP-positive subpopulation (Fig. 1A, B; lower panels) of all viable cells (gate R1), respectively. The specificity of virion entry and viral gene expression was confirmed by neutralization with an anti-VSV-G monoclonal antibody [24] or by pretreatment with the reverse transcriptase inhibitor efavirenz, respectively (Fig. 1; right panels).

**Figure 2:**
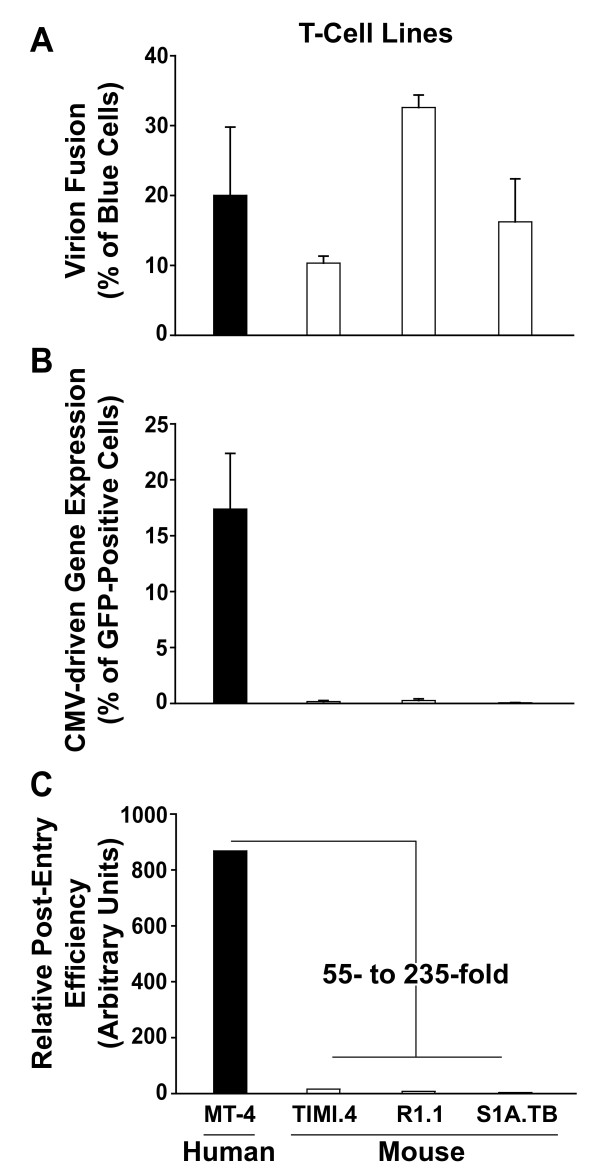
**Mouse T-cell lines of different genetic background allow VSV-G-mediated HIV-1 entry, but restrict CMV-driven gene expression**. Results for (A) virion fusion and (B) CMV-driven GFP gene expression for MT-4 (human), TIMI.4, R1.1 and S1A.TB (mouse) T-cell lines from the experiment shown in Fig. 1. Values are the arithmetic mean ± S.D. of triplicates. Panel C depicts the Relative-Post-Entry-Efficiency calculated as the ratio of the percentage of GFP-expressing cells (panel B) divided by the percentage of cleaved-CCF2-positive cells (virion fusion; panel A) × 1000 in arbitrary units. Data are representative for 3–4 independent experiments.

T-cell lines from both species allowed quite similar levels of entry of the BlaM-Vpr-loaded HIV-CMV-GFP virus (Figs. 1A, B; upper panels), ranging on average from 10 to 31% (Fig. 2A). In stark contrast, analysis of GFP reporter expression showed a 67- to 290-fold reduction in the percentage of infected mouse T-cell lines (TIMI.4; R1.1, S1A.TB) expressing the reporter transgene compared to human MT-4 T-cells (Figs. 1A, B; lower panels; Fig. 2B). This degree of impairment in gene expression was also seen when equal titres of VSV-G HIV-CMV-GFP, that did not carry BlaM-Vpr, were used, or when gene expression was assessed on day 7 p.i. (data not shown). In a more refined analysis, the ratio of the percentages of cells that scored positive for gene expression (Fig. 2B) relative to virion entry (Fig. 2A) was defined for each T-cell line as a cumulative Relative Post-Entry Efficiency revealing a mouse-human species differerence of 55- to 235-fold (Fig. 2C). In summary, these consecutive analysis of virion entry and CMV-driven reporter gene expression from a single infection corroborate the observation of a severe post-entry block for HIV-1 in mouse T-cell lines [20,21].

### HIV-1 reverse transcription and nuclear import occur efficiently in mouse T-cells

To characterize at which step of the replication cycle following entry HIV-1 encounters a block in murine T-cells, levels of late HIV-1 cDNAs and episomal 2-LTR circles were analyzed as markers for reverse transcription and nuclear import of the pre-integration complex, respectively. DNA was extracted from infected mouse and human T-cell lines (from the experiment shown in Figs. 1, 2), aliquots of which were harvested 24 h p.i. and analyzed by real-time PCR. The HIV-1 cDNA species were quantified using established protocols, specificity controls, and quantitative standards for either HIV-1 cDNA species and normalized to cellular DNA levels, which were determined in a parallel reaction by amplification of a cellular gene [6,25].

**Figure 3:**
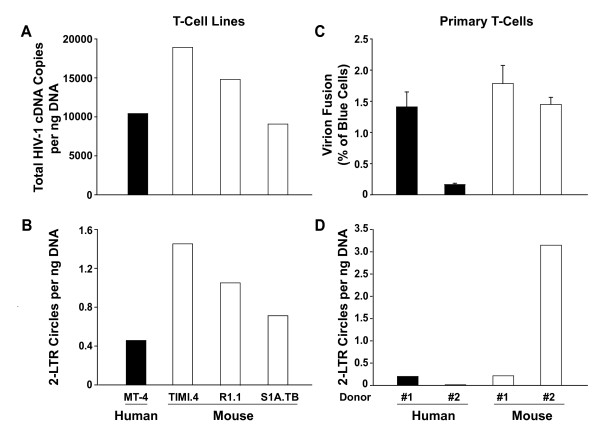
**HIV-1 reverse transcription and nuclear import of the pre-integration complex are well supported in T-cell lines and primary T-cells from mice following infection with VSV-G HIV-CMV-GFP**. (A, B) The levels of total HIV-1 cDNA and 2-LTR circles were quantified in infected human and mouse T-cell lines, samples being derived from the experiment shown in Figs. 1, 2. One day p.i. cell aliquots were taken and the extracted DNA analyzed for HIV-1 cDNA species and a species-specific gene by real-time PCR as described under *Methods*. Triplicate samples were analyzed and data are representative for three independent experiments. (C, D) Mitogen/IL-2-activated primary T-cells from two human donors or two pools of splenocytes from 3 BALB/c mice were infected with VSV-G HIV-CMV-GFP BlaM-Vpr and analyzed for (C) virion fusion or (D) 2-LTR circles 6 h and 1d p.i., respectively. Duplicate samples were analyzed.

Following comparable levels of virion entry (Fig. 2A), levels of total HIV-1 cDNA were found to be quite similar in the cross-species comparison (Fig. 3A), suggesting an efficient reverse transcription process in these rodent cells. Similarly, levels of episomal 2-LTR circles were in the same range or slightly elevated in mouse T-cell lines relative to the human counterpart (Fig. 3B). Following normalization with levels of *de novo *synthesized HIV-1 cDNA (Fig. 3A), 2-LTR circle levels (Fig. 3B) turned out to be statistically indistinguishable (data not shown). Importantly, 2-LTR circles were also detected in HIV-1-infected primary mouse T-cells derived from splenocyte pools of 3 BALB/c mice. Levels of 2-LTR circles were comparable (Fig. 3D; mouse donor pool #1) or ~16-fold higher (Fig. 3D; mouse donor pool #2) than in primary human T-cell cultures, indicating that following virion entry (Fig. 3C) the processes of reverse transcription and nuclear import are intact in these primary mouse targets. As a specificity control, no 2-LTR circles could be detected in efavirenz-treated cultures, demonstrating that the amplified episomal HIV-1 cDNAs had been generated from *de novo *synthesized viral DNA and were not present in the inoculum (data not shown). Due to the generally low infection level and residual DNase-resistant, production-related plasmid contaminations in virus stocks, levels of *de novo *synthesized viral DNA could not be quantified separately in primary T-cells (data not shown). In summary, these results suggest that following entry of virions, reverse transcription occurs efficiently in mouse T-cells. Furthermore, abundant levels of 2-LTR circles suggest robust import of the pre-integration complex into the nucleus. This tentatively maps the replication barrier in mouse T-cells to a step after nuclear entry.

### Establishment and validation of a quantitative nested PCR to detect integrated HIV-1 DNA in the mouse genome

**Figure 4:**
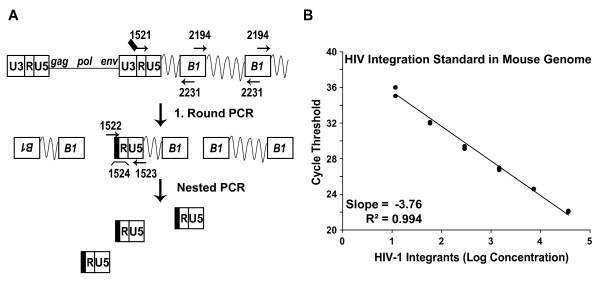
**Establishment of a quantitative PCR for the detection of HIV-1 integrants in the mouse genome**. (A) Schematic representation of the strategy and primers for the nested mouse integration PCR using anchor primers specific for highly repetitive mouse *B1 *elements. A mouse integration standard was generated by infection of mouse NIH3T3 fibroblasts with a low MOI of VSV-G HIV-1_NL4-3 _GFP, passaging of cells and sorting for GFP-expressing cells 7 weeks p.i. following trichostatin A induction. The resulting cell population was termed NIH3T3^int ^cells. (B) Representative HIV-1 integration standard amplification based on NIH3T3^int ^cells plotted as a function of the natural logarithm of the concentration of HIV-1 (log concentration) versus the values of the PCR cycle threshold. The correlation coefficient R^2 ^and the slope of the correlation are given.

Next, we quantified provirus formation in infected mouse and human T-cells. In principle, a defect at the level of integration can drastically diminish or completely abrogate viral gene expression [26,27]. Similar to reported nested PCR strategies to amplify HIV-1 integrated in proximity to highly abundant genomic repeat elements in human cells (*Alu *elements) [28], or in rat cells (*BC *elements) [6], we designed a nested real-time PCR to specifically quantify integrated HIV-1 provirus in mouse cells using the most abundant consensus sequence *B1 *within mouse *SINE *elements [29], as the repeat target for the cellular anchor primer pair in the genome of this species (Fig. 4A).

To establish a standard for quantitative analyses of integration into the mouse genome, a stable polyclonal population of NIH3T3 fibroblasts containing integrated HIV-1 provirus was generated by infection with VSV-G HIV-1_NL4-3 _GFP at a low MOI, cell passage for 7 weeks to allow complete loss of all unintegrated HIV-1 cDNA species, and subsequent enrichment of GFP-positive cells by flow cytometric sorting (thereafter referred to as NIH3T3P^int ^cells), in principle as reported previously for the rat species [6]. Since these NIH3T3P^int ^cells no longer contain unintegrated HIV-1 cDNA species, the absolute number of HIV-1 integrants was accurately quantified by the number of total HIV-1 cDNA copies (54 HIV-1 cDNA copies per ng DNA), thus providing a faithful reference for the integration PCR standard in the mouse genome. Fig. 4B depicts a typical mouse HIV-1 integration standard plotted as a function of the natural logarithm of the concentration of HIV-1 versus the PCR cycle threshold. This standard has a dynamic range of over 3 logs with a highest copy number of 36.741, and both the slope and R^2 ^value were considered as quality controls in individual experiments.

**Figure 5:**
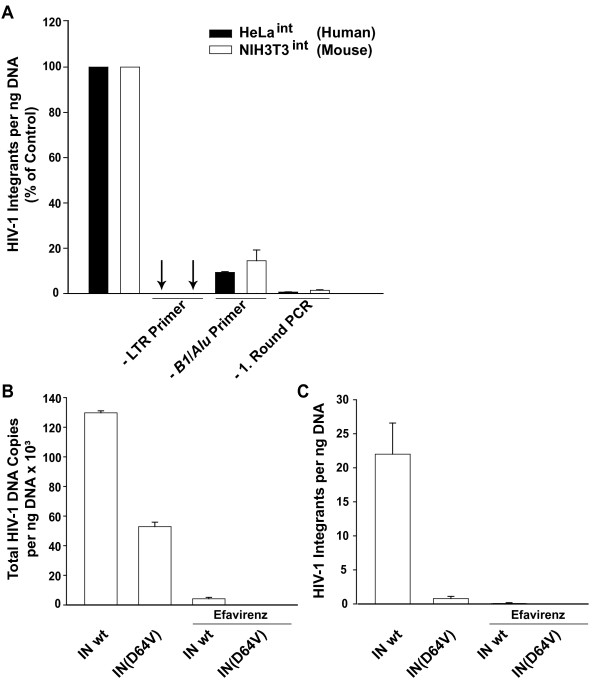
**Validation of the quantitative PCR for the detection of HIV-1 integrants in the mouse genome**. (A) Technical validation of species-specific HIV-1 integration PCRs on genomic DNA from mouse NIH3T3^int ^cells or human HeLa^int ^cells (20). Levels of HIV-1 integrants from the complete standard PCR reaction were set to 100% and levels determined for several specificity controls are given relative to that (omission (-) of LTR primer #1521, omission (-) of cellular anchor primer pair (*B1*, #2194 and #2231 (mouse) or *Alu*, #1519 and #1520 (human), omission (-) of first round PCR reaction). (B, C) Validation of the mouse integration PCR in a dynamic infection context. Parental NIH3T3 cells were infected with VSV-G HIV-CMV-GFP, carrying either a wildtype integrase (IN wt) or a catalytically inactive integrase mutant (IN(D64V)). Where indicated, the NNRTI efavirenz was added 1 h prior to infection. (B) Infected NIH3T3 cells were monitored for levels of total HIV-1 cDNA on day 1 p.i. (C) On day 7 p.i., cells were analyzed for the presence of integrated HIV-1 cDNA applying the mouse integration PCR.

The nested PCR strategy for quantification of HIV-1 integrants in mouse cells is depicted in Fig. 4A, and described in the figure legend and under *Methods*. This mouse integration PCR and a human integration PCR, the latter essentially following a published protocol [28], were validated side-by-side using genomic DNA from NIH3T3^int ^or HeLa^int ^cells [6], respectively (Fig. 5A). Here, the number of HIV-1 integrants per ng DNA for the complete PCR reaction was set to 100% for each species. First, omission of LTR primer #1521 from the first-round PCR reaction resulted in a loss of the amplification signal in both species. Second, a reaction mix without the cellular anchor primer pair (Fig. 5A; #2194 and #2231 (mouse); or #1519 and #1520 (human), [6]) yielded low residual signals, most likely due to the partial formation of single-stranded DNA from LTR-containing HIV-1 cDNA by the first-round LTR primer, as reported [6,28]. Finally, omission of the first-round PCR reaction did not give a signal above background (Fig. 5A), indicating that second-round amplification of non-preamplified DNA is not of significant concern.

Next, the mouse integration PCR was further validated in a dynamic infection context. Levels of total HIV-1 cDNA and integrants were quantified in parental NIH3T3 fibroblasts infected with either the integration-competent (IN wt) HIV-CMV-GFP or an integration-defective isogenic HIV-1 vector (IN(D64V)), the latter carrying a catalytic core mutation in integrase [30]. One day after infection, high levels of total HIV-1 cDNA were found in NIH3T3 cells challenged with either lentiviral vector, while only background levels could be amplified from efavirenz-treated controls (Fig. 5B). In DNA extracted on day 7 p.i., integrants were readily amplified by the newly developed real-time PCR protocol in NIH3T3 cells infected with the IN wt vector, while the level of provirus formation was severely reduced with the IN(D64V) mutant (Fig. 5C). Collectively, a real-time PCR for the specific detection and absolute quantification of HIV-1 integrants in the genome of infected mouse cells was established and validated.

### Levels of HIV-1 integrants are severely reduced in infected mouse T-cell lines

**Figure 6:**
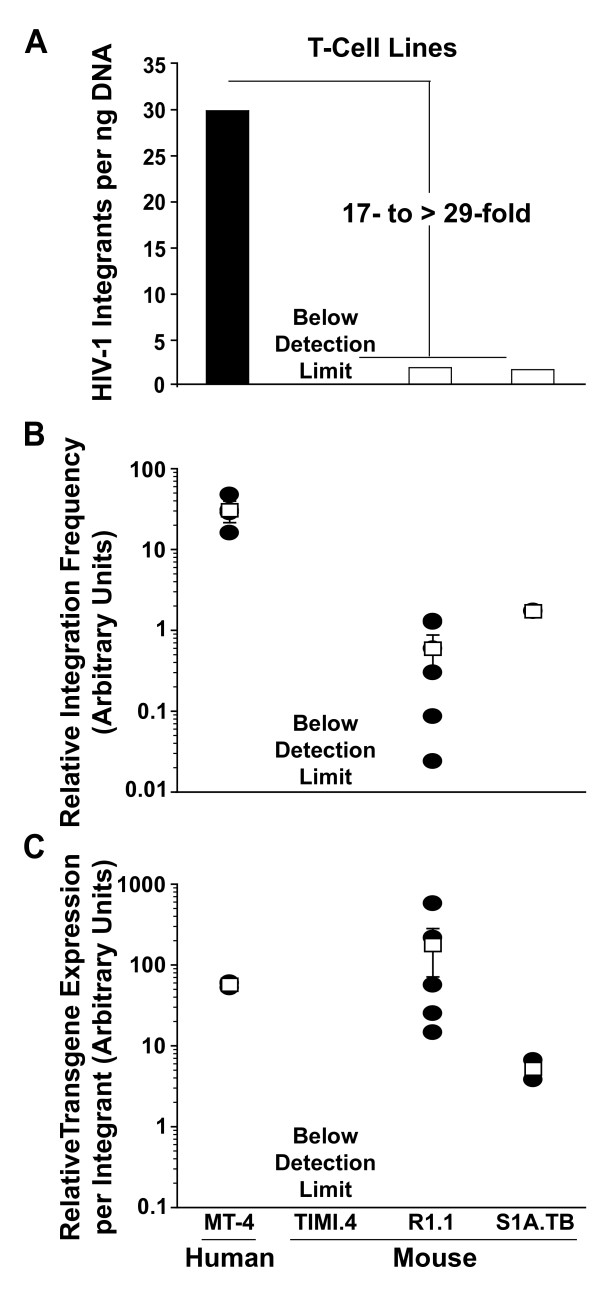
**HIV-1 integration is inefficient in mouse T-cell lines**. (A) Human and mouse T-cell lines, infected with VSV-G HIV-CMV-GFP (see also results in Figs. 1–3), were analyzed for levels of HIV-1 integrants using species-specific integration PCRs (Figs. 4, 5 and [6]). Triplicate samples were analyzed. (B) The Relative-Integration-Frequency was calculated as the number of integrants on day 7 p.i. (Fig. 6A) relative to the total amount of HIV-1 cDNA on day 1 p.i. (Fig. 3A) for the identical T-cell infection. -(C) Furthermore, the relative CMV-driven Transgene- Expression-per-Integrant was deduced from the identical experiments and calculated as the ratio of the percentage of GFP-positive cells on day 3 p.i. (Fig. 2B) relative to the number of integrants per ng cellular DNA on day 7 p.i. (Fig. 6A). In (B) and (C) the resulting ratio × 100 is given in arbitrary units, and the arithmetic means ± S.E.M. of 3–5 independent experiments each with duplicates or triplicates are shown.

Next, we applied the mouse integration PCR to a dynamic VSV-G HIV-CMV-GFP infection in mouse T-cell lines. While virion entry (Fig. 2A), reverse transcription (Fig. 3A), and nuclear import (Fig. 3B) appeared to be intact in infected mouse T-cells, HIV-1 integrants from the identical experiment were undetectable in the genome of TIMI.4 cells and reduced 17- to > 29-fold in R1.1 and S1A.TB cells relative to human MT-4 T-cells (Fig. 6A). To establish a signature characteristic for individual T-cell lines in respect to their ability to allow HIV-1 integration, we calculated the Relative-Integration-Frequency defined as the number of integrants on day 7 p.i. (Fig. 6A) relative to the total amount of HIV-1 cDNA quantified on day 1 p.i. (Fig. 3A). Based on results from 2–5 independent experiments, infected TIMI.4 cells were grossly impaired in their Relative-Integration-Frequency value, > 305-fold compared to infected MT-4 cells (Fig. 6B). The two other mouse T-cell lines, R1.1 and S1A.TB, displayed an intermediate phenotype with Relative-Integration-Frequency values 18- to 54-fold lower compared to the human reference. Thus, integration into the host cell genome appears to be the paramount barrier imposed by mouse T-cells in the early phase of HIV-1 replication.

### Evidence for cyclin T1-independent transcriptional deficit in certain T-cell lines

To gain insight into the quantitative relationship between vector integrants and transgene expression, the ratio of the percentage of T-cells expressing GFP from the CMV IE promoter and levels of integrants per cell was calculated, for convenience termed Transgene-Expression-per-Integrant. For the residual integrants in mouse R1.1 T-cells (Fig. 6A, B), reporter gene expression was efficient, resulting in a Transgene-Expression-per-Integrant value in a range similar to human MT-4 cells (Fig. 6C). In contrast, the Transgene-Expression-per-Integrant value in infected S1A.TB cells was 34-fold lower than R1.1 (Fig. 6C), indicating that integrants in this mouse T-cell line frequently do not result in a detectable gene expression. This suggests that at least in some T-cell lines a defect in transgene expression from residual integrants may impose an additional peri-integrational limitation in the mouse species.

### Following infection with a near-full length HIV-1_NL4-3_, mouse T-cells do not support early viral gene expression

**Figure 7:**
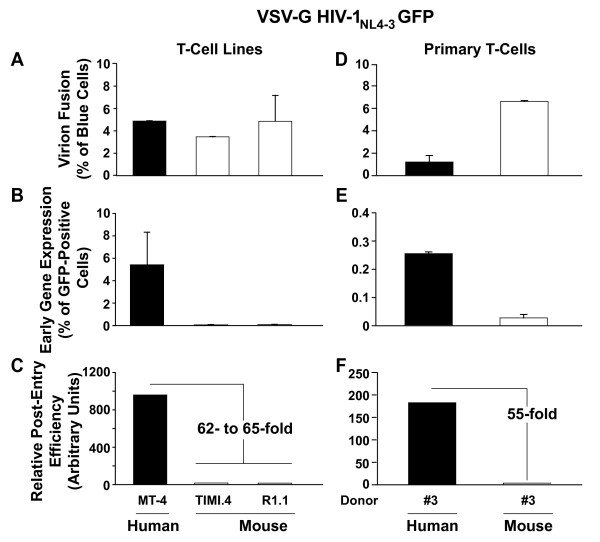
**Mouse T-cells do not support early viral gene expression following infection with a near-full length HIV-1_NL4-3_**. (A-C) T-cell lines and (D-F) primary T-cells of mouse and human origin were challenged with VSV-G pseudotyped HIV-1_NL4-3 _GFP virions carrying BlaM-Vpr and analyzed for virion fusion and early HIV-1 gene expression in principle as described in the legends to Figs. 1, 2. Shown are the arithmetic means ± S.D. Panels C and F depict the Relative- Post-Entry-Efficiency calculated as described in the legend to Fig. 2.

Finally, we sought to confirm the key findings also in the context of an infection with a near-full length HIV-1_NL4-3_. This replication-deficient, envelope-deleted molecular proviral clone carries an *egfp *reporter gene within the *nef *locus driven by the 5'-LTR, and virions were also VSV-G pseudotyped and loaded with BlaM-Vpr during production. Following infection with this virus, the Relative Post-Entry Efficiency in TIMI.4 and R1.1 mouse T-cells was 62- to 65-fold lower compared to human MT-4 (Fig. 7C) or Jurkat T-cells (data not shown), and thus in a range similar to that seen for the lentiviral vector (55- to 235-fold; Fig. 2C), despite the cyclin T1-dependence of gene expression. Of note, the post-entry restriction was not overcome at an MOI > 5, determined by saturating virion fusion conditions in the respective mouse T-cell lines (data not shown). Importantly, relative to T-cells from a human donor, a pool of primary T-cells from BALB/c mice also displayed a severely reduced Relative-Post-Entry-Efficiency following infection with VSV-G HIV-1_NL4-3 _GFP (55-fold; Fig. 7F).

**Figure 8:**
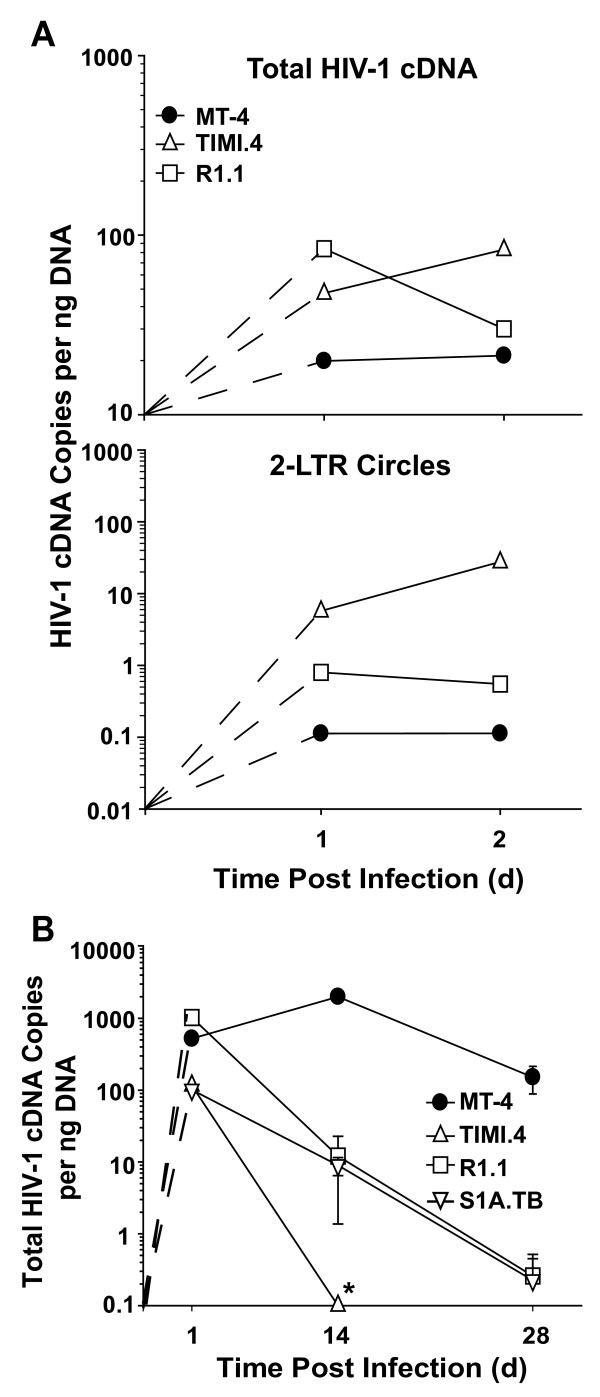
**HIV-1 reverse transcription and nuclear import of the pre-integration complex are well supported in mouse T-cell lines after infection with nearly full-length HIV-1_NL4-3_**. (A) The relative levels of (upper panel) total HIV-1 cDNA, and (lower panel) 2-LTR circles were quantified in human (MT-4) and mouse (TIMI.4, R1.1) T-cell lines infected with VSV-G HIV-1_NL4-3 _GFP on day 1 and 2 p.i., and samples derived from the experiment shown in Figs. 7A-C. At the indicated time points p.i. cell aliquots were taken and DNA analyzed by real-time PCR for the respective HIV-1 cDNA species. Duplicate samples were quantified. (B) Dynamic of HIV-1 cDNA load in passaged T-cell lines. Cells infected with VSV-G HIV-1_NL4-3 _GFP BlaM-Vpr were cultivated for four weeks. The relative levels of total HIV-1 cDNA shown are derived from DNA analyses of two independent infections from cell samples taken on days 1, 14, and 28 p.i. *below detection limit.

Furthermore, in cell aliquots removed on day 1 and 2 p.i. from the experiment shown in Figs. 7A–C, levels of total HIV-1 cDNA were quite similar in the cross-species comparison with infected mouse T-cell lines harbouring 1.4- to 4-fold higher levels than human MT-4 T-cells (Fig. 8A, upper panel). Most notably, levels of episomal 2-LTR circles were found to be drastically elevated in TIMI.4 T-cells (51- to 245-fold) and modestly enhanced in R1.1 T-cells (5- to 7-fold) relative to the human counterpart (Fig. 8A, lower panel). Following normalization with levels of *de novo *synthesized HIV-1 cDNA, 2-LTR circle levels were statistically indistinguishable for R1.1 and MT-4 T-cells. For reasons unknown, the DNase-resistant, production-related plasmid contamination in stocks of VSV-G HIV-1_NL4-3 _GFP was typically 2–3 orders of magnitude higher compared to VSV-G HIV-CMV-GFP, and, as a consequence, integration could not be reliably quantified for the former virus in T-cells from either species (data not shown).

In an attempt to circumvent this technical limitation, we reasoned that the reduced ability of mouse T-cells to form proviruses should be reflected in a loss of cell-associated HIV-1 cDNA during prolonged passage. Consequently, cells were challenged with the replication-defective VSV-G HIV-1_NL4-3_GFP carrying BlaM-Vpr and continuously cultivated for four weeks. First, levels of virus entry (6 h p.i.; data not shown), *de novo *synthesized HIV-1 cDNA (Fig. 8B), and 2-LTR circles (data not shown) were fairly comparable on 1 day p.i., confirming the intact early phase of the replication cycle and successful nuclear import of the preintegration complex. In contrast, a massive loss of cell-associated HIV-1 cDNA was observed in all three mouse T-cell lines at day 14 p.i. (Fig. 8B; 102- to > 2317-fold reduction) with levels in passaged TIMI.4 cells at background. On day 28 p.i., levels of amplified HIV-1 cDNA were further reduced in R1.1 and S1A.TB cells. Collectively, these results provide additional evidence that HIV-1 cDNA, despite nuclear localization, cannot efficiently integrate into the genome of mouse T-cell lines, resulting in a largely abortive infection.

## Discussion

The mapping of HIV-1 replication barriers in non-human cells has allowed the identification and molecular characterization of critical host factors [3,31] and potent restriction factors [32,33]. This has, on one hand, expanded our understanding of fundamental processes in the host-virus interaction and, on the other hand, fuelled efforts to develop genetically altered rodents and non-human primates that are highly permissive for HIV-1 [1,14,34,35]. In the current study we demonstrate that T-cells from mice restrict HIV-1 replication at the level of integration.

We examined the fate of the virion and viral genome through consecutive steps in the early infection phase following a single challenge with dual-reporter HIV-1 virions. To circumvent potential HIV-1 Tat/cyclin T1-dependent, species-specific differences in LTR transactivation [3], we used an HIV-based vector encoding for GFP driven by a CMV promoter. This experimental approach corroborated the finding reported in three earlier studies that primary and immortalized T-cells from mice harbor a severe post-entry restriction for HIV-1, in the context of infections with virions carrying either HIV envelopes or VSV-G [19-21]. Of note, analogous experiments using a near-full length HIV-1_NL4-3_, in which the GFP reporter is driven off the 5'-LTR (Fig. 7), demonstrated a similar defect in the Relative-Post-Entry-Efficiency in mouse T-cells.

Carefully controlled virion fusion and real-time PCR analyses showed that reverse transcription per fused HIV-1 particle is at least as efficient in T-cells from mice as in their human counterparts. Furthermore, relative levels of 2-LTR circles were comparable or in some instances even markedly elevated in mouse T-cells (Figs. 3D, 8A), demonstrating transfer of the pre-integration complex into the nucleus. In contrast, Baumann *et al*. [20] reported reduced efficiencies at both of these replication steps in the identical mouse T-cell lines used in our study. The reason for these discrepancies is currently unclear. In this report [20] we noted several differences to our study, including (i) a side-by-side comparison of infected mouse T-cells only with mouse NIH3T3 fibroblasts, but not human T-cells, (ii) a lack of correlative evaluations of consecutive replication steps, (iii) a lack of control for residual proviral plasmid contamination in virus inocula, and (iv) quantification of HIV-1 cDNA species without a PCR-based normalization for corresponding cell equivalents.

In line with our observations, Tsurutani *et al*. [21] found that reverse transcription proceeds normally in mouse T-cells and that levels of 2-LTR circles were similar in the cross-species comparison following an HIV-1 IN wt infection. Using a cassette ligation-mediated PCR these authors noted a qualitative reduction in integrants in primary lymphocytes from mice. However, based on 2- to 8-fold lower relative 2-LTR circle levels for infections with an integrase-defective HIV-1, Tsurutani *et al*. located the restriction exclusively at the level of nuclear import in mouse cells. Conceivably, the abundance of 2-LTR circles can be affected by a large number of parameters including the levels of reverse transcribed viral DNA, the efficiency of nuclear import of the pre-integration complex, the efficiency of integration, the activity of cellular ligases of the non-homologous DNA end joining pathway, the formation frequency of other episomal HIV-1 cDNA species (1-LTR circles and auto-integrants), the degradation kinetics of episomes, the time point p.i. as well as the frequency of cell division [36-39]. Of note, the ratio of 2-LTR circles per total HIV-1 cDNA was ~100- to 1000-fold lower in the identical T-cell lines infected with the lentiviral vector (0.008% – 0.01%; Fig. 2A, B) compared with the near full-length HIV-1_NL4-3 _virus, 0.6%; 1.2% and 25.6% (see Fig. 8A, B). In addition to the difficulties in assessing 2-LTR circle levels discussed above, this also suggests marked construct-dependent differences in the formation of these HIV-1 cDNA episomes. While the presence of 2-LTR circles can be taken as unambiguous proof for nuclear transfer of pre-integration complexes, a quantitative assessment of this episomal DNA species, in light of the plethora of factors influencing its steady-state levels, in our view, does not allow a selective examination and conclusive interpretation of the efficiency of nuclear import in a cross-species comparison.

As a technical advance, we established a nested real-time PCR to specifically detect integrants in the mouse genome. Of note, repetitive *B1 *elements in the mouse genome are approximately 5-fold less abundant than *Alu *elements in the human genome (2% versus 10%) [40], which potentially renders the mouse HIV-1 integration PCR slightly less sensitive. This does, however, not affect the quantification of absolute integrant numbers per genome equivalent, since each species-specific HIV-1 integration standard was calibrated against a total HIV-1 cDNA quantification in long-term passaged NIH3T3^int ^and HeLa^int ^cells. This strategy thus allows a direct comparison of absolute levels of integrants between two species.

First and most importantly, we identify and quantitatively describe a severe limitation at the level of provirus formation in infected mouse T-cell lines, in which pre-integration complexes had entered the nucleus. Based on the above arguments, we cannot formally exclude a quantitative limitation in nuclear import in mouse T-cells based on the 2-LTR circle analyses, however, we can clearly demonstrate a paramount defect at the level of integration. Different degrees of integration impairment were noted with TIMI.4 cells displaying the most drastic restriction, while Relative-Integration-Frequency values in R1.1 and S1A.TB cells were 18- to 54-fold lower compared to human MT-4 cells. As an important characteristic, the barrier was not overcome at high MOIs which favors the hypothesis of a lacking supportive cellular factor rather than the presence of an inhibitory or restriction factor. Furthermore, analysis of the Transgene-Expression-per-Integrant suggests that a cyclin T1-independent transcriptional defect may impose an additional peri-integrational limitation for a productive HIV-1 infection at least in some mouse T-cell lines. Conceptually, integration into transcriptionally unfavorable sites in the host genome [41,42] could result in such a phenotype.

The fate of HIV-1 cDNA following nuclear entry is a step in the retroviral replication cycle that only recently is being recognized to be dependent on and modulated by specific host factors. Most notably, lense epithelium-derived growth factor (LEDGF/p75) is a chromatin-associated host protein that directly interacts with integrase and apparently tethers the pre-integration complex to the chromosome [43-47]. LEDGF/p75 is, on one hand, essential for an efficient integration process [27,48] and, on the other hand, likely involved in the targeting of HIV-1 integration into transcription units in human cells [49,50]. Both of these characteristics make LEDGF/p75 a potential candidate for the observed peri-integrational deficits in mouse T-cells. Interestingly, murine and human LEDGF/p75 are highly homologous and all known functional regions are 100% conserved. Embryonal fibroblasts from *Psip1/Ledgf *knockout mice displayed severely reduced levels of HIV-1 vector integration [42]. Of note, in this isogenic mouse context, a ~10-fold reduction in HIV-1 integrants coincided with a modest, ~1.5-fold increase in 2-LTR circles, consistent with the relative constellation seen for these two HIV-1 cDNA species in the cross-species comparison in the majority of our experiments. Furthermore, a critical role of mouse LEDGF/p75 in gene-specific integration was demonstrated in null cells both by mapping of residual integration sites and the observation of reduced transgene expression levels per integrant [42]. Consequently, it could be highly informative to determine and potentially manipulate levels of endogenous LEDGF/p75 in restricted mouse T-cells.

Additional cellular proteins of interest include emerin and barrier-to-autointegration factor, which have been proposed to contribute to the appropriate nuclear localization of the viral DNA for chromatin engagement prior to integration in human cells [26], although this is still controversial [51]. Of potential relevance for a scenario involving an inhibitory activity, Zhang *et al*. identified p21^Waf1/Cip1/Sdi1 ^as a cellular factor that can influence the sensitivity of human hematopoietic precursors for infection by HIV-1 at a post-entry step [52]. Specifically, expression of p21 in these human cells altered the fate of HIV-1 cDNA in the nucleus, apparently promoting the formation of episomal 2-LTR circles at the expense of integration.

Mice and rats are the prime candidates for the development of an immunocompetent, multi-transgenic small animal model. Primary target cells from both rodents share the inability to support virion entry and efficient transcription, and these limitations can be overcome by transgenic expression of the HIV-1 receptor complex [1,2,35] or human cyclin T1 ([6,14], and C.G. and O.T.K., unpublished observation), respectively. Contrasting the severe early-phase restriction in mouse T-cells, rat T-cells efficiently support all steps of the HIV-1 replication cycle up to and including integration [6]. Thus, mice appear to impose at least one additional replication barrier compared to rats which highlights the complexity of the task to develop genetically altered mice that are fully permissive for HIV-1 infection. The here identified restriction at the level of integration in mouse T-cells will facilitate the identification of critical host factors towards this goal.

## Methods

### Animals

Female BALB/c mice were obtained from Charles River Laboratories (Sulzfeld, Germany). Animals were kept in the central animal facility of the University of Heidelberg.

### Cells

The mouse T lymphoma cell lines S1A.TB.4.8.2 (S1A.TB) (TIB-27; BALB/c strain), TIMI.4 (TIB-37; C57BL/6 strain) and R1.1 (TIB-42; C58.J strain) were obtained from the American Type Culture Collection and cultivated in Dulbecco's modified Eagle medium supplemented with 10% fetal calf serum or horse serum (R1.1), 1% penicillin-streptomycin, and 1% L-glutamine (all from GIBCO). Human T-cell lines MT-4 and Jurkat, the adherent cell lines TZM-bl, 293T, HeLa^int^, and mouse fibroblast cell line NIH3T3 cells (CRL-1658; NIH/Swiss strain) were cultivated as reported [6,7,53-55]. Primary T-cells from Ficoll-gradient purified peripheral blood mononuclear cells from healthy human donors were activated by phytohaemagglutinin-P (1 μg/ml) and human recombinant interleukin-2 (IL-2) (20 nM, BioMol, Germany) as reported [25,35,53,56]. Primary T-cells from BALB/c mice were prepared from spleens, which had been removed aseptically from sacrificed animals. Single-cell suspensions were prepared by pushing tissue pieces through a nylon mesh screen (70-μm-pore-size nylon, Becton Dickinson). The cell suspension was washed once with PBS and erythrocytes were lysed for 3 min with ACK lysis buffer, followed by another PBS wash. Single-cell splenocyte suspensions were cultivated in supplemented medium and activated with Concanavalin A (1 μg/ml) and IL-2 as reported [56], yielding proliferating cultures containing ~90% CD3-positive T-cells.

### DNA extraction

Cellular and viral DNAs were extracted using Qiagen DNeasy Tissue Kits^®^. Samples for quantitative HIV-1 integration PCR were extracted according to the method of Hirt [57,58]. Briefly, 5 × 10^5 ^cells were pelleted and carefully resuspended in 160 μl HIRT Solution I. Next, 10 μl Proteinase K (20 mg/ml) and 200 μl HIRT Solution II were added and the cells were incubated for 30 min at 56°C. Then, 100 μl of 5 M sodium chloride were added to the reaction prior to overnight incubation at 4°C. Afterwards, chromosomal DNA was pelleted and the supernatant removed. The HIRT pellets were then extracted with phenol-chloroform-isoamyl alcohol (25:24:1), ethanol precipitated in the presence of glycogen, and washed in 70% ethanol prior to resuspending the DNA pellet in elution buffer (Qiagen).

### Virus stocks

VSV-G pseudotyped stocks of a three-plasmid HIV-1-based GFP vector [59] (HIV-CMV-GFP) or of HIV-1_NL4-3 _E^- ^GFP [60], carrying BlaM-Vpr, were generated in 293 T cells by calcium phosphate DNA precipitation in principle as described [6,7]. Virus stocks were characterized for p24 CA concentration by antigen enzyme-linked immunosorbent assay and for infectious titer on TZM-bl cells, respectively [53]. Virus stocks were treated with DNase (Turbo DNase, Ambion, Dresden, Germany) for 1 h at 37°C (5 IU per 10 μl of concentrated virus stock) to reduce the degree of plasmid contamination prior to challenge of target cells.

### HIV-1 virion-fusion assay and gene expression analysis

Target T-cells (5 × 10^6^) were left untreated or pretreated with efavirenz (100 μM, Bristol-Myers Squibb, Uxbridge, UK) for 1 h, or a hybridoma supernatant containing anti-VSV-G monoclonal antibody I1 [24] for 15 min at 37°C. Subsequently, cells were infected with BlaM-Vpr-loaded VSV-G HIV-1 for 6 h, washed and stained overnight with CCF2/AM dye. Fusion was monitored with a three-laser BD FACSAria Cell Sorting System (Becton Dickinson, San Jose, CA) as reported [6,22,23,25]. On day 3 p.i., the percentage of GFP-positive cells was measured in the identical cultures on a FACSCalibur using BD CellQuest Pro 4.0.2 Software (BD Pharmingen).

### Quantification of total HIV-1 cDNA and 2-LTR circles

Levels of total HIV-1 cDNA and 2-LTR circles in infected cell lines were measured by quantitative PCR using the ABI 7500 sequence detection system (Applied Biosystems, Foster City, CA) as reported in detail [25]. For PCR standard curves, dilutions of pHIV-1_NL4-3 _and pU3U5, respectively, covering 5 logs were used, supplemented with DNA extracted from uninfected cells. Results for HIV-1 cDNA species were normalized to the amount of cellular DNA, which was quantified in a parallel amplification of the mouse GAPDH gene or human RNaseP gene (Applied Biosystems), respectively. Genomic standards were derived from dilutions of genomic DNA extracted from uninfected cells. All samples were run in duplicate. Data analysis was performed with the 7500 System Software (Applied Biosystems). Of note, for all HIV-1 cDNA analyses values obtained for parallel infections of efavirenz-treated cultures were subtracted from values of untreated cultures to account for residual proviral plasmid contamination in DNAse-treated virus inocula. For total HIV-1 cDNA analyses shown in this manuscript for VSV-G HIV-CMV-GFP (Figs. 1, 2, 3, 4, 5, 6) or VSV-G HIV-1_NL4-3 _GFP (Figs. 7, 8), the subtracted signals ranged between 0.07 – 0.65% or 0.3 – 4.6% of the untreated infection, respectively. However, in several experiments with other VSV-G HIV-1_NL4-3 _GFP stocks this background signal reached levels above 60% (despite DNAse treatment), not allowing a reliable quantification of total HIV-1 cDNA or integrants.

### Quantification of integrated HIV-1 cDNA

The strategy to quantify HIV-1 integrants in the mouse genome by real-time PCR and the generation of a mouse integration standard cell line, NIH3T3^int ^cells, was adapted from the protocol established for the rat species [6]. Briefly, HIV-1 integrants were amplified in the first reaction by one primer annealing to the LTR (primer #1521 [28]), which contains a lambda-phage heel sequence at the 5'-end, and by two outward-facing primers that target the highly redundant consensus sequence within the mouse *B1 *gene [40] (primers #2194, 5'-ACAGCCAGGGCTACACAGAG-3' and #2231, 5'-CCTCCCAAGTGCTGGGATTAAAG-3'). The first-round amplicon was then amplified in a second reaction with a lambda-specific primer (primer #1522 [28]) and an LTR primer (primer #1523, [6]) and an LTR-specific probe (probe #1524, [6]). The second-round cycling conditions were identical to those used to determine the total amounts of HIV-1 cDNA and 2-LTR circles [25]. For the detection of HIV-1 integrants in infected human cells, the conditions and reagents were identical to those reported [6]. For each integration PCR, a specificity control reaction was run in parallel in which the cellular primer pair was omitted during the first round reaction and this value was subtracted from the total signal (not more than 20% of the absolute signal). For integration standard curves, dilutions of genomic DNA derived from NIH3T3^int ^and HeLa^int ^cells covering 3.1 logs were used (see also Fig. 4B). Criteria for standard validity included the slope, which indicates the efficiency of the PCR reaction within a defined copy range using diluted cellular DNA carrying defined amounts of integrated provirus (ideally 3.32, corresponding to a 100% efficient reaction). In addition, only integration PCR assays in which the standard reached a R^2 ^coefficient > 0.9 were analyzed. The lowest detection standard of the PCR was 0.04 and 0.12 HIV-1 integrants per ng DNA for integrated provirus in mouse and human genomic DNA, respectively.

## Competing interests

The authors declare that they have no competing interests.

## Authors' contributions

HMT, CG and OTK designed the study. HMT conducted all of the experiments. CG provided input on the virion-fusion assay and on the establishment and validation of the mouse integration PCR. HMT and OTK wrote the paper. All authors commented on and approved the final manuscript.
